# Relationship between Red Blood Cell Indices (MCV, MCH, and MCHC) and Major Adverse Cardiovascular Events in Anemic and Nonanemic Patients with Acute Coronary Syndrome

**DOI:** 10.1155/2022/2193343

**Published:** 2022-11-03

**Authors:** Zhanyi Zhang, Shanshan Gao, Mengya Dong, Jian Luo, Chenbo Xu, Wen Wen, Yuzhi Huang, Yue Wu, Juan Zhou, Zuyi Yuan

**Affiliations:** ^1^Department of Cardiovascular Medicine, The First Affiliated Hospital of Xi'an Jiaotong University, Xi'an, Shaanxi, China; ^2^Key Laboratory of Environment and Genes Related to Diseases, Ministry of Education, Xi'an Jiaotong University, Xi'an, Shaanxi, China; ^3^Key Laboratory of Molecular Cardiology, Xi'an Jiaotong University, Xi'an, Shaanxi, China; ^4^Department of Cardiology, Shaanxi Provincial People's Hospital, Xi'an, Shaanxi, China; ^5^Health Management Center, Xi'an People's Hospital (Xi'an Fourth Hospital), Xi'an, Shaanxi, China

## Abstract

**Background:**

Previous studies have shown that increased mean corpuscular volume (MCV) is an independent predictor for worse outcomes in coronary artery disease. However, as parameters to classify different types of anemia together with MCV, the relationship between mean corpuscular hemoglobin (MCH), mean corpuscular hemoglobin concentration (MCHC), and long-term outcomes in acute coronary syndrome (ACS) remains obscure. Moreover, few studies have compared the prognostic value of these red blood cell indices in anemic and nonanemic patients with ACS.

**Methods and Results:**

In this single-center observational cohort study, we enrolled 393 patients diagnosed with ACS, including 75 anemic and 318 nonanemic patients. The composite end points were defined as major adverse cardiovascular events (MACEs). After a median follow-up of 31.24 months, Kaplan–Meier survival analysis showed that higher MCV and MCH but not MCHC were significantly associated with increased MACEs in nonanemic ACS patients. Among the enrolled ACS patients without anemia, Cox regression analysis revealed that high MCV and MCH were correlated with increased MACEs after adjustment for cardiovascular risk factors, and receiver operating characteristic (ROC) curve analysis further confirmed the predictive value of high MCV and MCH. In bivariate correlation and linear regression analysis, plasma homocysteine was positively correlated with MCV and MCH but not MCHC in the nonanemic group even after adjusting for age, male sex, BMI, SBP, DBP, smoking, and diabetes. However, MCV, MCH, and MCHC showed no predictive value for MACEs, and no correlation was found between these red blood cell indices and homocysteine in ACS patients with anemia.

**Conclusion:**

After adjusting for cardiovascular risk factors, this study showed that higher admission MCV and MCH but not MCHC were independent predictors for long-term MACEs and positively correlated with homocysteine levels in the blood among the nonanemic but not anemic patients with ACS in China.

## 1. Introduction

Cardiovascular disease (CVD) is the leading cause of death worldwide, and the prevalence and mortality of CVD have increased significantly in China during the last 2 decades [1]. Acute coronary syndrome (ACS) is a serious form of CVD with a high mortality risk. With the development of early diagnosis, the number of patients with relatively moderate types of ACS in the Chinese population is remarkably increasing [2]. Therefore, it is necessary to find parameters that are routinely assessed, easily attainable, and inexpensive to help us improve the diagnosis and prognosis of ACS.

Mean corpuscular volume (MCV), mean corpuscular hemoglobin (MCH), and mean corpuscular hemoglobin concentration (MCHC) are red blood cell indices included in routine blood examination which is widely used in clinic. It has been reported that high MCV was correlated with high mortality in patients with acute myocardial infarction (AMI) [3], acute decompensated heart failure [4], and consecutive patients subjected to PCI [5]. Our previous study also showed that increased MCV is an independent predictive factor for major adverse cardiovascular events (MACEs) in ACS patients after stent implantation [6]. However, the relationship between MCH, MCHC, and long-term outcomes in ACS patients is still obscure.

It is well known that anemia is independently associated with increased all-cause mortality in ACS patients [7]. Although MCV, MCH, and MCHC are closely related to anemia, the differences between the prognostic values of these red cell indices in anemic and nonanemic ACS patients are still unclear. Therefore, the present study was conducted to investigate the relationship between these red blood cell indices and long-term MACEs in ACS patients with or without anemia.

## 2. Materials and Methods

### 2.1. Study Design

**Figure 1:**
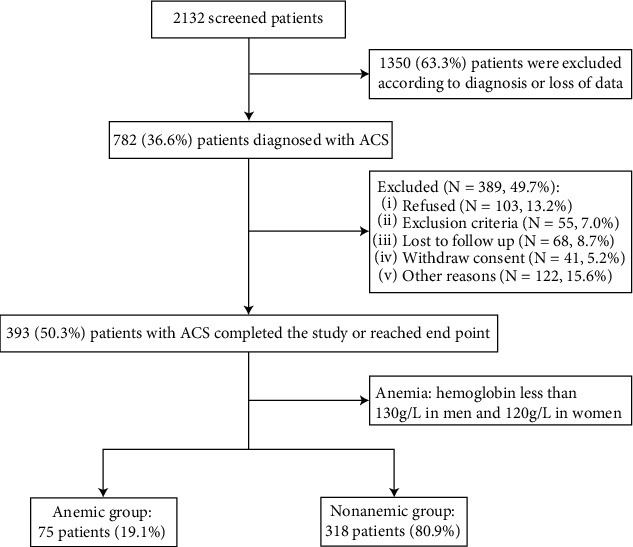
Flowchart of the present clinical study. ACS: acute coronary syndrome.

The current study is a retrospective analysis of a single-center prospective cohort study. After screening of 2132 consecutive patients diagnosed of coronary artery disease at the First Affiliated Hospital of Xi'an Jiaotong University from January 2013 to February 2014, a total of 393 patients diagnosed with ACS were enrolled in this research and completed the follow-up. Then, these patients were divided into anemic (*n* = 75) and nonanemic (*n* = 318) groups according to the level of hemoglobin (male < 130 g/L and female < 120 g/L), which is the definition provided by the World Health Organization [8]. The study flowchart is shown in Figure 1. The inclusion criteria were a diagnosis of ACS, which was defined as unstable angina (UA), non-ST-segment elevation myocardial infarction (NSTEMI), and ST-segment elevation myocardial infarction (STEMI) according to the guidelines [9]. In addition to loss of MCV, MCH, or MCHC values, the exclusion criteria were heart failure, hematologic disorders (except for anemia), malignant tumors, infections, liver or renal dysfunction, autoimmune diseases, pregnancy, and certain nutritional restrictions or gastrointestinal diseases that lead to erythrocyte maturation disorders. Patients who were alcoholic and had used drugs such as diuretics that could lead to erythrocyte index disturbances were also excluded. This study was carried out in accordance with the Declaration of Helsinki and approved by the Ethics Committee of Xi'an Jiaotong University, and all participants provided written informed consent.

Baseline characteristics, including demographic data, laboratory tests, primary diagnosis, and discharge medication, were recorded from medical records. Traditional cardiovascular risk factors consisted of age, sex, body mass index (BMI), smoking, diabetes, systolic blood pressure (SBP), diastolic blood pressure (DBP), left ventricular ejection fraction (LVEF), low-density lipoprotein cholesterol (LDL-C), CKMB, homocysteine, high-sensitivity C-reactive protein (hsCRP), and N-terminal pro-brain natriuretic peptide (NT-proBNP).

### 2.2. Follow-Up and Outcome

MACEs, as composite end points, were defined as all-cause death, cardiac death, acute myocardial infarction, urgent revascularization, rehospitalization, and cerebrovascular diseases (including stroke, transient ischemic attacks, and cerebral infarction) [10]. The follow-up visits were carried out through telephone or personal interviews by well-trained physicians until March 24, 2016, and the median follow-up time was 31.24 months for 393 patients with ACS. Both anemic and nonanemic patients were divided into 3 groups according to MCV, MCH, and MCHC tertiles.

### 2.3. Laboratory Measurements

To make sure that the biochemical parameters were collected at a standardized baseline and to eliminate the potential interference of confounding factors such as circadian rhythms, food intake, and the general condition of patients, peripheral vein blood samples were obtained from each patient early in the morning the day after admission when they had already fasted for 12 hours overnight. Then, samples were drawn into EDTA anticoagulation tubes (BD Biosciences, USA) and used for biochemical measurements. Routine blood examination, including MCV, MCH, and MCHC, was measured with an automatic hematology analyzer (Sysmex 2100, Japan) 30 minutes after sample collection. LVEF and other biochemical parameters were measured using standard methods.

### 2.4. Statistical Analysis

Continuous data were analyzed for normality using the Kolmogorov–Smirnov test. Data are presented as mean ± standard deviations for normally distributed data or medians (25-75th percentiles) for nonnormally distributed data. Categorical data are expressed as counts (percentages). Two-tailed unpaired Student's *t*-test and Mann–Whitney *U* test were used to compare the data between two groups. One-way ANOVA and Kruskal–Wallis analysis were used to determine the statistical significance among multiple groups. Categorical variables were compared using the chi-square test between different groups. Kaplan–Meier survival analysis and log-rank tests were performed to compare the adverse event-free survival rate between each group. Cox regression analysis was applied to calculate HRs and 95% confidence intervals (CIs) to determine the independent predictors of MACEs. Receiver-operating characteristic (ROC) curve analysis was used to evaluate the ability of MCV, MCH, and MCHC to predict adverse clinical events. Spearman correlation and partial correlation analyses were used to determine the correlation between MCV, MCH, and MCHC and cardiovascular risk factors. Simple linear regression analysis was used to examine the correlation between homocysteine and MCV, MCH, and MCHC levels in the blood. All the data were analyzed using SPSS 18.0 (SPSS Inc., Chicago, Illinois), and a two-sided *p* value < 0.05 was considered statistically significant.

## 3. Results

### 3.1. Baseline Characteristics of ACS Patients with and without Anemia

A total of 393 patients (304 men and 89 women, mean age: 60.4 ± 10.2 years) who completed follow-up and reached the end points were enrolled in this study and divided into anemic (*n* = 75) and nonanemic (*n* = 318) groups according to different levels of hemoglobin (Figure 1). The baseline characteristics of the total, anemic, and nonanemic groups are listed in Table 1. Here, we showed that ACS patients with anemia were older, included more females, and had higher NT-proBNP levels, but the BMI, DBP, ALT, LDL-C, and usage of ACEIs/ARBs were decreased compared with the nonanemic group. MCH, MCHC, hemoglobin, and HCT were significantly lower, while the level of RDW-CV was higher in anemic ACS patients. Notably, coronary revascularization during admission, number of lesion vessels, and implanted stents showed no difference in anemic and nonanemic groups. The incidence of MACEs in the anemic group (26.7%) was significantly higher than that in the nonanemic (14.5%) group (*p* = 0.011). No significant differences were observed in other baseline parameters (all had *p* values > 0.05).

### 3.2. Baseline Characteristics Stratified by MCV, MCH, and MCHC Tertiles

The anemic and nonanemic patients with ACS were divided into three groups based on MCV, MCH, and MCHC levels. Baseline characteristics are shown in Supplementary Table 1, Supplementary Table 2, and Supplementary Table 3.

In the anemic group, we found that the number of patients with diabetes was significantly lower in the highest tertile of MCV and MCH, and LDL-C was lower with increased levels of MCH. RDW-CV was decreased, and the usage of ACEIs/ARBs was increased in higher tertiles of MCHC. The MACE occurrence showed no significance among groups with different MCV, MCH, and MCHC levels in ACS patients with anemia (all had *p* values > 0.05).

In the nonanemic group, with increasing levels of MCV, MCH, and MCHC, the number of male and smoking patients also increased. The number of diabetes cases significantly decreased in groups with higher MCV and MCH. We also found that homocysteine was remarkably higher in the highest MCV and MCHC groups but showed no significant change in different MCHC tertiles. ALT and hemoglobin were elevated in higher tertiles of MCH and MCHC. HCT was increased in higher tertiles of MCV, and the level of RDW-CV was reduced while MCHC was increased. The use of coronary revascularization showed no significant difference among the different tertiles of MCV, MCH, and MCHC. The incidence of MACEs in the highest tertile of MCV (23.1%) and MCH (23.0%) was higher than that in any other group (*p* = 0.009 for MCV and *p* = 0.013 for MCH), but no significant difference in MACEs was found in MCHC tertiles. Data regarding bleeding events and other baseline factors showed no significant difference in the anemic or nonanemic groups (all had *p* values > 0.05).

### 3.3. Kaplan–Meier Survival Analysis

**Figure 2:**
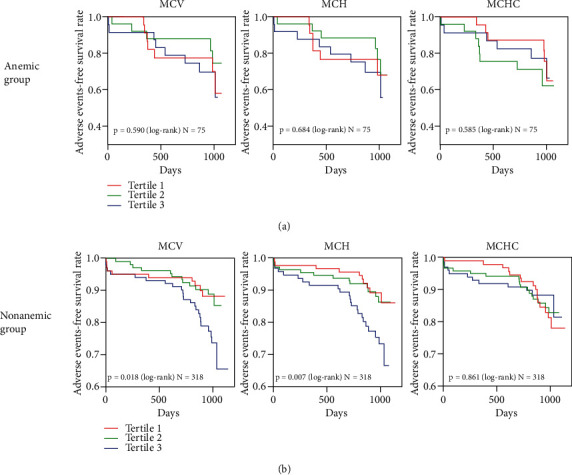
Kaplan–Meier survival analysis of MACEs based on MCV, MCH, and MCHC levels in ACS patients with or without anemia. (a) Adverse event-free survival rate for MACEs in 75 anemic patients divided by tertiles of MCV, MCH, and MCHC levels during the follow-up period. (b) Adverse event-free survival rate for MACEs in 318 nonanemic patients divided by tertiles of MCV, MCH, and MCHC levels during the follow-up period. ACS: acute coronary syndrome; MACEs: major adverse cardiovascular events; MCH: mean corpuscular hemoglobin; MCHC: mean corpuscular hemoglobin concentration; MCV: mean corpuscular volume.

Kaplan–Meier survival analysis was conducted to determine the long-term prognostic value of MCV, MCH, and MCHC in ACS patients with or without anemia (Figure 2). With a median follow-up of 31.24 months, 20 (26.7%) anemic and 46 (14.5%) nonanemic patients experienced MACEs. In the anemic group, we found no significant association between different levels of MCV, MCH, and MCHC and the risk of MACEs (Figure 2(a)). However, our results showed that higher MCV and MCH were significantly associated with increased MACEs (log-rank test: *p* = 0.018 for MCV and *p* = 0.007 for MCH). The highest tertile had the lowest adverse event-free survival rate in the nonanemic group (Figure 2(b)). There was no significant difference between MCHC and MACEs in ACS patients without anemia (Figure 2(b)).

### 3.4. Univariate and Multivariate Cox Regression Analysis

As shown in Table 2, we performed Cox regression analysis to further determine the relationship between these red cell indices and MACEs. Univariate analysis showed that high MCV (HR 2.347, 95% CI 1.121-4.913, *p* = 0.024) and MCH (HR 2.626, 95% CI 1.249-5.520, *p* = 0.011) were significantly associated with increasing MACEs in the nonanemic group. However, no significant association was found between these red cell indices and increased MACEs in the anemic group. We further performed a multivariate analysis of nonanemic patients. We found that high MCV (HR 2.419, 95% CI 1.103-5.305, *p* = 0.028) and MCH (HR 3.522, 95% CI 1.475-8.410, *p* = 0.005) were significantly associated with an increased risk of MACEs after adjusting for age, sex, diabetes, hypertension, smoking, and LVEF. This association remained significant even after further adjusting for RBC, hemoglobin, HCT, and RDW-CV (MCV: HR 2.743, 95% CI 1.184-6.355, *p* = 0.019; MCH: HR 4.530, 95% CI 1.808-11.348, *p* = 0.001).

### 3.5. Receiver-Operating Characteristic (ROC) Curve Analysis

**Figure 3:**
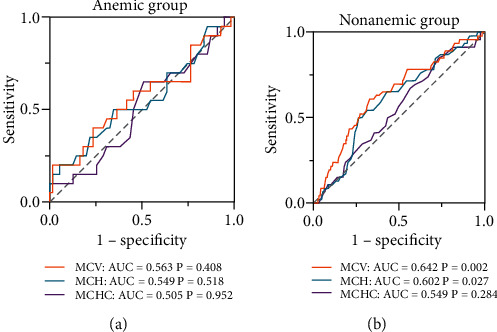
Receiver-operating characteristic (ROC) curve analysis in the anemic and nonanemic group. (a) ROC curve analysis of MCV, MCH, and MCHC in the anemic group. (b) ROC curve analysis of MCV, MCH, and MCHC in the nonanemic group. AUC: area under the curve; MCH: mean corpuscular hemoglobin; MCHC: mean corpuscular hemoglobin concentration; MCV: mean corpuscular volume.

To analyze the diagnostic ability of plasma MCV, MCH, and MCHC levels to predict the risk of MACEs, an ROC curve analysis was conducted (Figure 3). In anemic patients, no significant association was found for these red cell indices to predict MACEs (Figure 3(a)). The area under the curve (AUC) for MACEs was 0.642 (*p* = 0.002) for MCV and 0.602 (*p* = 0.027) for MCH in nonanemic ACS patients, and no association was found between MCHC and long-term MACEs in ROC analysis (Figure 3(b)).

### 3.6. Correlation between MCV, MCH, MCHC, and Cardiovascular Risk Factors

To investigate the association between MCV, MCH, MCHC, and cardiovascular risk factors, we performed Spearman and partial bivariate correlation analyses (Tables 3 and 4). As shown in Table 3, MCV and MCH were negatively correlated with LVEF and MCHC was negatively associated with SBP in anemic patients. In the nonanemic group, MCHC was negatively correlated with age. It is worth noting that homocysteine was the only biochemical parameter that was significantly positively correlated with MCV (*r* = 0.312, *p* < 0.001) and MCH (*r* = 0.287, *p* < 0.001) in the nonanemic group. Moreover, the positive correlation between homocysteine and MCV (*R* = 0.472, *p* < 0.001) and MCH (*R* = 0.405, *p* < 0.001) remained significant even after adjustment for age, male sex, BMI, SBP, DBP, smoking, and diabetes in the nonanemic group (Table 4).

### 3.7. Linear Regression Analysis between MCV, MCH, MCHC, and Homocysteine

**Figure 4:**
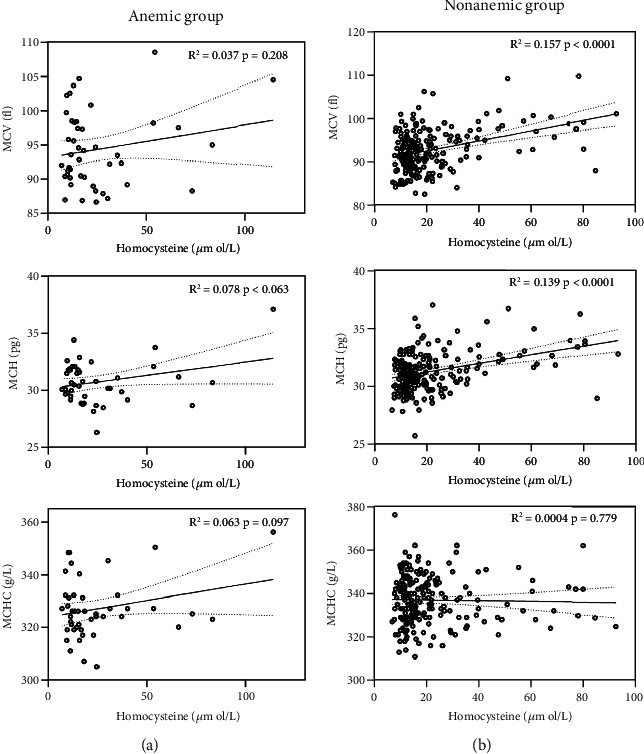
Linear regression analysis between homocysteine and MCV, MCH, and MCHC levels in anemic and nonanemic patients with ACS. (a) Linear regression of the relationship between homocysteine and MCV, MCH, and MCHC in ACS patients with anemia. (b) Linear regression of the relationship between homocysteine and MCV, MCH, and MCHC in ACS patients without anemia. ACS: acute coronary syndrome; MCH: mean corpuscular hemoglobin; MCHC: mean corpuscular hemoglobin concentration; MCV: mean corpuscular volume.

Linear regression analysis was performed to further investigate the correlation between these red blood cell indices and homocysteine in anemic and nonanemic ACS patients (Figure 4). There was no association between homocysteine and these red cell indices in the anemic group (Figure 4(a)). However, circulating homocysteine levels were positively correlated with MCV (*r*2 = 0.157, *p* < 0.0001) and MCH (*r*2 = 0.139, *p* < 0.0001) but were not correlated with MCHC in the nonanemic group (Figure 4(b)). Collectively, our study implied that homocysteine was a crucial factor that could have a possible connection to the detrimental effect of high MCV and MCH in nonanemic patients with ACS.

### 3.8. The Prognostic Value of MCV and MCH in UA/NSTEMI and STEMI Patients

Since culprit lesions may be different in UA/NSTEMI and STEMI patients, we further analyzed the ability of MCV and MCH to predict MACEs in these 2 groups separately. In this study, we showed that high MCV (log-rank test: *p* = 0.012) and MCH (log-rank test: *p* = 0.031) were significantly correlated with increased MACEs in UA/NSTEMI but not in STEMI patients, and ROC curve analysis exhibited that MCV (AUC = 0.637, *p* < 0.001) was significantly associated with poorer outcomes in the UA/NSTEMI group (Supplementary Figure 1). Then, we analyzed the predictive value of MCV and MCH among the anemic and nonanemic UA/NSTEMI patients and found that these red blood cell indices showed no predictive values in anemic patients, while high MCV (log-rank test: *p* = 0.028) and MCH (log-rank test: *p* = 0.018) were correlated with increased MACEs in those without anemia (Supplementary Figure 2).

## 4. Discussion

The aim of this study was to compare the relationship between MCV, MCH, and MCHC and long-term MACEs in anemic and nonanemic patients with ACS. The primary findings were as follows: (1) after adjustment for several classic cardiovascular risk factors, high MCV and MCH but not MCHC were independently associated with an increased incidence of long-term MACEs in nonanemic ACS patients but not in those with anemia; and (2) plasma homocysteine levels were significantly positively correlated with MCV and MCH but not MCHC in nonanemic patients with ACS even after adjustment for cardiovascular risk factors.

MCV, MCH, and MCHC are termed red cell indices included in routine blood examination to define the size and hemoglobin content of red blood cells [11]. MCV determines the size of red blood cells, MCH defines the amount of hemoglobin per red blood cell, and MCHC indicates the amount of hemoglobin per unit volume, which is calculated as MCH divided by MCV [11, 12]. It has been well described that a high MCV is correlated with worse outcomes in ACS patients [5, 6]. However, evidence regarding the prognostic values of MCH and MCHC in coronary artery disease is scarce and contradictory [13, 14]. In the present study, we confirmed the independent predictive value of high MCV and MCH but not MCHC for long-term MACEs in a population of nonanemic ACS patients in China. Elevated MCV and MCH levels can both be used as indicators for macrocytosis [15]. As a morphological and functional dysfunction of red blood cells, macrocytosis is present in approximately 60% of patients without associated anemia [16]. It could lead to an imbalance between the cytoplasm and nucleus, hinder the flow of less flexible red blood cells through microcirculation, and impair the antioxidant properties of the erythrocyte membrane [17, 18]. Although macrocytosis is unlikely to show any obvious clinical signs or symptoms [15], our study emphasizes that we still need to pay special attention to nonanemic ACS patients with high MCV or MCH levels. However, among patients with high MCHC, the smaller size and higher hemoglobin content per cell not only make it easier for red blood cells to pass through tiny capillaries but also relatively increase oxygen supply to the heart [19], which could be the reason why we did not find a correlation between high MCHC and worse outcomes in ACS patients regardless of whether anemia was present.

Anemia is present in 10.5% to 46.4% of patients admitted to the hospital for ACS [20]. Macrocytic anemia had the highest mortality in patients undergoing stent implantation among the 3 major subtypes of anemia stratified by MCV level [21]. However, in STEMI patients with anemia, another study found that MCV was not an independent predictor for in-hospital bleeding and mortality, and the worse outcome was not determined by the type of anemia but by atrial fibrillation, older age, diabetes, and other chronic noncardiovascular illnesses [22]. In this study, we also found no significant association between MCV, MCH, MCHC, and long-term MACEs in ACS patients with anemia. As the most common type of anemia worldwide, microcytic hypochromic anemia induced by iron deficiency is characterized by low MCV, MCH, and MCHC [23], which may normalize the increased levels of these red cell indices and cover up the pathological mechanism of macrocytosis in anemic patients [15]. Moreover, during acute and chronic anemia, the diameter and volume of capillary vessels in the heart were significantly increased to compensate for the reduced oxygen-carrying capacity of the blood [24, 25]. Therefore, in anemic patients with high MCV and MCH levels, enlarged red blood cells can pass through capillaries more smoothly than those without anemia, which could counteract the detrimental effect of macrocytosis on ACS patients.

Previous studies have found a positive correlation between red blood cell distribution width (RDW) and homocysteine in the general population and patients with STEMI [26–28]. In this study, we provided a novel finding that even after adjustment for age, male sex, BMI, SBP, DBP, smoking, and diabetes, plasma homocysteine was also significantly positively correlated with MCV and MCH levels in nonanemic ACS patients. Elevated homocysteine is a classic proatherogenic risk factor associated with an increased risk of MACEs among ACS patients [29]. Homocysteine can initiate and exacerbate atherosclerosis by multiple pathophysiological mechanisms such as stimulating endothelial dysfunction, platelet generation, vascular smooth muscle cell proliferation, and reactive oxygen species production [30]. In addition, homocysteine could also dose-dependently enhance the procoagulant activity of red blood cells by exposure to phosphatidylserine and the generation of microparticles [31]. However, the underlying mechanisms of this correlation between high homocysteine levels and the morphological and functional impairment of red blood cells are still obscure and require further investigation. Increased oxidative stress, associated with macrocytosis and high homocysteine levels in the blood, may play indispensable roles [18, 32].

Because UA/NSTEMI and STEMI patients exhibited different clinical features, we also analyzed the predictive value of these red blood cell indices in these two groups of patients. In this study, we showed that high MCV and MCH were significantly correlated with an increased risk of MACEs in UA/NSTEMI but not in STEMI patients, and these red blood cell indices were only correlated with increased MACEs in those without anemia. A previous study (*n* = 248) has already found that high MCV is an independent predictor of poorer clinical outcomes in nonanemic patients with acute myocardial infarction [3]. However, the study population was relatively small (*n* = 64) and there were only 6 patients with STEMI who experienced MACEs; the results may not be entirely convincing in the STEMI patients of this study. Therefore, after careful consideration, we decided to put this part of the results in the Supplementary Materials and will continue to investigate the prognostic value of these red blood cell indices in UA/NSTEMI and STEMI patients in our future studies.

There are several limitations of this study. First, this is a single-center study with a relatively small sample size, and more extensive multicenter clinical research is required to provide more information. Second, the observational cohort design could not draw causal relationships between these red blood cell indices, homocysteine, and clinical outcomes and inevitably has confounding bias. Third, the exact mechanisms through which MCV and MCH impact long-term outcomes could not be elucidated. Data on the levels of folate and vitamin B12 and analysis of iron deficiency parameters in the blood would significantly contribute to the quality of this work. In addition, the patient's therapy on discharge is mentioned in this study but not before admission to the hospital, and MCV, MCH, and MCHC levels should be detected during follow-up. Last but not least, due to the time of blood sample collection, this study only recruited patients who did not die before the early morning the day after admission and we have excluded those who have heart failure or renal dysfunction. Therefore, the results from this study may not be suitable for ACS patients with high-risk complications, such as cardiac arrest, shock, cardiac dysfunction, and renal failure.

## 5. Conclusions

In conclusion, high MCV and MCH but not MCHC were correlated with increased long-term MACEs in nonanemic but not anemic ACS patients, and elevated homocysteine in the blood could be correlated with the detrimental effect of high MCV and MCH on nonanemic ACS patients. As standard and easily obtained laboratory parameters, measuring these red blood cell indices could be helpful to predict the long-term clinical outcomes in ACS patients, especially for those without anemia.

## Figures and Tables

**Table 1:** Baseline characteristics in total, anemic, and nonanemic patients with acute coronary syndrome.

	Total (*n* = 393)	Anemic group (*n* = 75)	Nonanemic group (*n* = 318)	*p* value
*Demographics and risk factors*				
Age (year)	60.4 ± 10.3	66.3 ± 10.3	59.0 ± 9.7	<0.001
Male	306 (77.9%)	49 (65.3%)	257 (80.8%)	0.004
Body mass index (kg/m^2^)	25.0 ± 3.2	24.2 ± 2.8	25.2 ± 3.3	0.038
SBP (mmHg)	122.5 (117.3-140.0)	125.0 (115.0-140.0)	120.0 (118.5-140.0)	0.894
DBP (mmHg)	80.0 (70.0-83.8)	74.0 (70.0-80.0)	80.0 (70.0-85.0)	0.002
Smoking	224 (57.0%)	33 (44.0%)	191 (60.1%)	0.011
Diabetes	83 (21.1%)	22 (29.3%)	61 (19.2%)	0.053
Family history	163 (41.5%)	25 (33.3%)	138 (43.4%)	0.112
Past MI	63 (16.0%)	7 (9.3%)	56 (17.6%)	0.079
Past PCI or CABG	82 (20.9%)	19 (25.3%)	63 (19.8%)	0.290
Bleeding events	14 (3.6%)	5 (6.7%)	9 (2.8%)	0.107
*Laboratory tests*				
MCV (fL)	93.1 (90.3-96.3)	93.5 (90.5-98.4)	93.1 (90.3-95.9)	0.149
MCH (pg)	31.1 (30.0-32.3)	30.7 (29.4-32.1)	31.3 (30.2-32.3)	0.028
MCHC (g/L)	334.0 (325.0-342.5)	324.0 (319.0-332.0)	336.0 (328.0-344.0)	<0.001
LVEF (%)	63.0 (51.0-68.0)	63.0 (51.8-70.3)	62.0 (51.0-68.0)	0.434
Aspartate aminotransferase (U/L)	26.4 (18.7-49.0)	27.0 (18.8-54.7)	26.2 (18.7-48.9)	0.725
Alanine aminotransferase (U/L)	24.9 (17.1-43.1)	20.3 (14.9-35.3)	27.1 (18.0-46.5)	0.018
Creatinine (*μ*mol/L)	66.4 (57.9-76.1)	65.8 (57.2-76.2)	66.4 (58.2-76.0)	0.893
LDL-C (mmol/L)	2.1 (1.7-2.7)	2.0 (1.5-2.5)	2.2 (1.7-2.8)	0.009
CKMB (U/L)	15.0 (11.3-26.6)	14.3 (11.1-27.1)	15.2 (11.4-25.9)	0.532
Homocysteine (*μ*mol/L)	16.1 (12.2-24.6)	16.2 (11.8-29.2)	16.0 (12.2-24.0)	0.756
RBC (10^12^/L)	4.7 (3.7-6.6)	4.1 (3.1-6.6)	4.7 (3.8-6.6)	0.053
Hemoglobin (g/L)	138.9 ± 17.3	114.0 ± 13.2	144.7 ± 12.3	<0.001
HCT (%)	41.5 ± 4.7	35.0 ± 3.9	43.1 ± 3.4	<0.001
RDW-CV (%)	13.2 (12.6-13.7)	13.5 (12.9-14.5)	13.1 (12.6-13.6)	<0.001
hsCRP (mg/dL)	1.4 (0.7-3.6)	1.6 (0.6-3.7)	1.4 (0.7-3.5)	0.844
NT-proBNP (pg/mL)	340.5 (116.0-891.5)	467.0 (171.5-1542.0)	312.3 (112.2-792.1)	0.010
*Primary diagnosis at admission*				
UA	293 (74.6%)	59 (78.7%)	234 (73.6%)	
NSTEMI	36 (9.2%)	4 (5.3%)	32 (10.1%)	
STEMI	64 (16.3%)	12 (16.0%)	52 (16.4%)	
*In-hospital treatment*				
Coronary revascularization	387 (98.5%)	74 (98.7%)	313 (98.4%)	0.879
Number of lesion vessels	3.0 (2.0-3.0)	3.0 (2.0-3.0)	3.0 (2.0-3.0)	0.460
Number of stents	2.0 (1.0-3.0)	2.0 (1.0-3.0)	2.0 (1.0-3.0)	0.901
*Discharge medication*				
Aspirin	389 (99.0%)	73 (97.3%)	316 (99.4%)	0.114
Clopidogrel	385 (98.0%)	72 (96.0%)	313 (98.4)	0.180
Ticagrelor	19 (4.8%)	5 (6.7%)	14 (4.4%)	0.411
Beta-blocker	346 (88.0%)	64 (85.3%)	282 (88.7%)	0.422
ACEI/ARB	359 (91.3%)	64 (85.3%)	295 (92.8%)	0.039
Statin	386 (98.2%)	75 (100.0%)	311 (97.8%)	0.195
Calcium channel blocker	89 (22.6%)	19 (25.3%)	70 (22.0%)	0.537
*MACEs*	66 (16.8%)	20 (26.7%)	46 (14.5%)	0.011
All-cause death	17 (4.3%)	9 (12.0%)	8 (2.5%)	<0.001
Cardiac death	11 (2.8%)	7 (9.3%)	4 (1.3%)	<0.001
Acute myocardial infarction	6 (1.5%)	3 (4.0%)	3 (0.9%)	0.052
Urgent revascularization	18 (4.6%)	3 (4.0%)	15 (4.7%)	0.789
Rehospitalization	29 (7.4%)	10 (13.3%)	19 (6.0%)	0.028
Cerebrovascular diseases	9 (2.3%)	2 (2.7%)	7 (2.2%)	0.808

Data are shown as mean ± SD, median (interquartile range), or number (%). ACEI: angiotensin converting enzyme inhibitor; ARB: angiotensin receptor blocker; CABG: coronary artery bypass grafting; CKMB: creatine kinase, MB isoenzyme; DBP: diastolic blood pressure; HCT: hematocrit value; hsCRP: high sensitivity C-reactive protein; LDL-C: low-density lipoprotein cholesterol; LVEF: left ventricular ejection fraction; MACEs: major adverse cardiovascular events; MCH: mean corpuscular hemoglobin; MCHC: mean corpuscular hemoglobin concentration; MCV: mean corpuscular volume; MI: myocardial infarction; NSTEMI: non-ST elevated myocardial infarction; NT-proBNP: N-terminal pro-B-type natriuretic peptide; PCI: percutaneous coronary intervention; RBC: red blood cell; RDW-CV: red cell volume distribution width-coefficient of variation; SBP: systolic blood pressure; STEMI: ST elevated myocardial infarction; UA: unstable angina.

**Table 2:** Multivariate Cox regression analysis for MACEs of MCV, MCH, and MCHC.

	Anemic group			Nonanemic group								
	Unadjusted			Unadjusted			Model 1			Model 2		
	HR	95% CI	*p* value	HR	95% CI	*p* value	HR	95% CI	*p* value	HR	95% CI	*p* value
*MCV*												
Tertile 1	1.0 (ref)			1.0 (ref)			1.0 (ref)			1.0 (ref)		
Tertile 2	0.605	0.192-1.907	0.391	1.075	0.464-2.489	0.866	1.127	0.479-2.648	0.785	1.253	0.521-3.011	0.615
Tertile 3	1.039	0.376-2.870	0.941	2.347	1.121-4.913	0.024	2.419	1.103-5.305	0.028	2.743	1.184-6.355	0.019
*p* for trend			0.597			0.022			0.033			0.029
*MCH*												
Tertile 1	1.0 (ref)			1.0 (ref)			1.0 (ref)			1.0 (ref)		
Tertile 2	0.755	0.243-2.343	0.626	1.134	0.497-2.587	0.765	1.268	0.531-3.026	0.593	1.477	0.607-3.593	0.390
Tertile 3	1.204	0.417-3.473	0.732	2.626	1.249-5.520	0.011	3.522	1.475-8.410	0.005	4.530	1.808-11.348	0.001
*p* for trend			0.688			0.010			0.004			0.001
*MCHC*												
Tertile 1	1.0 (ref)			1.0 (ref)			1.0 (ref)			1.0 (ref)		
Tertile 2	1.694	0.585-4.906	0.332	0.925	0.471-1.815	0.820	0.895	0.441-1.819	0.760	0.871	0.311-2.441	0.793
Tertile 3	1.148	0.370-3.562	0.811	0.812	0.384-1.719	0.586	0.815	0.374-1.772	0.605	0.762	0.135-4.288	0.758
*p* for trend			0.591			0.862			0.873			0.953

Model 1: adjusted for age, male, diabetes, hypertension, smoking, and LVEF. Model 2: further adjusted for RBC, hemoglobin, HCT, and RDW-CV. 95% CI: 95% confidence interval; HCT: hematocrit value; HR: hazard ratio; LVEF: left ventricular ejection fraction; MACEs: major adverse cardiovascular events; MCH: mean corpuscular hemoglobin; MCHC: mean corpuscular hemoglobin concentration; MCV: mean corpuscular volume; RBC: red blood cell; RDW-CV: red cell volume distribution width-coefficient of variation; ref: reference group.

**Table 3:** Spearman correlation analysis of MCV, MCH, and MCHC with cardiovascular risk factors.

Variable	Anemic group						Nonanemic group					
	MCV		MCH		MCHC		MCV		MCH		MCHC	
	*r*	*p* value	*r*	*p* value	*r*	*p* value	*r*	*p* value	*r*	*p* value	*r*	*p* value
*Anthropometric characteristics*												
Age (year)	0.163	0.162	0.182	0.118	-0.046	0.693	0.067	0.237	-0.079	0.160	-0.182	0.001
BMI (kg/m^2^)	-0.138	0.320	-0.061	0.659	0.134	0.333	-0.095	0.129	-0.041	0.514	0.046	0.468
*Traditional risk factors*												
SBP (mmHg)	0.037	0.751	0.131	0.261	-0.230	0.048	-0.035	0.537	-0.005	0.934	0.038	0.505
DBP (mmHg)	0.096	0.414	0.158	0.177	0.167	0.152	-0.077	0.171	-0.009	0.869	0.070	0.213
*Echocardiographic and biochemical parameters*												
LVEF (%)	-0.263	0.028	-0.269	0.025	-0.175	0.147	0.072	0.217	0.012	0.832	-0.111	0.057
LDL-C (mg/dL)	-0.178	0.132	-0.220	0.062	-0.013	0.915	-0.006	0.911	-0.050	0.384	-0.082	0.154
CKMB (U/L)	0.156	0.182	0.175	0.133	0.164	0.161	0.071	0.209	0.083	0.142	0.007	0.896
Homocysteine (*μ*mol/L)	-0.022	0.887	-0.115	0.452	-0.015	0.923	0.312	<0.001	0.287	<0.001	-0.013	0.851
hsCRP (mg/dL)	0.227	0.099	0.239	0.082	0.093	0.504	-0.084	0.220	-0.068	0.326	0.074	0.282
NT-proBNP (pg/mL)	0.079	0.508	0.082	0.492	-0.024	0.837	-0.023	0.695	-0.010	0.862	0.038	0.515

BMI: body mass index; CKMB: creatine kinase, MB isoenzyme; DBP: diastolic blood pressure; hsCRP: high sensitivity C-reactive protein; LDL-C: low-density lipoprotein cholesterol; LVEF: left ventricular ejection fraction; MCH: mean corpuscular hemoglobin; MCHC: mean corpuscular hemoglobin concentration; MCV: mean corpuscular volume; NT-proBNP: N-terminal pro-B-type natriuretic peptide; SBP: systolic blood pressure.

**Table 4:** Partial correlation analysis of MCV, MCH, and MCHC with cardiovascular risk factors.

Variable	Anemic group						Nonanemic group					
	MCV		MCH		MCHC		MCV		MCH		MCHC	
	*r*	*p* value	*r*	*p* value	*r*	*p* value	*r*	*p* value	*r*	*p* value	*r*	*p* value
*Echocardiographic and biochemical parameters*												
LVEF (%)	-0.145	0.565	-0.151	0.549	-0.009	0.972	0.133	0.195	0.054	0.602	-0.111	0.283
LDL-C (mg/dL)	-0.161	0.522	-0.236	0.347	-0.125	0.621	0.087	0.399	0.006	0.956	-0.118	0.253
CKMB (U/L)	0.437	0.070	0.145	0.566	-0.406	0.094	-0.063	0.544	-0.151	0.141	-0.145	0.159
Homocysteine (*μ*mol/L)	0.146	0.564	0.455	0.058	0.445	0.064	0.472	<0.001	0.405	<0.001	-0.101	0.329
hsCRP (mg/dL)	0.260	0.297	0.181	0.471	-0.090	0.722	-0.068	0.512	-0.027	0.795	0.060	0.562
NT-proBNP (pg/mL)	0.056	0.825	0.144	0.570	0.126	0.619	0.065	0.532	0.087	0.398	0.032	0.760

Adjusted for age, male, BMI, SBP, DBP, smoking, and diabetes. BMI: body mass index; CKMB: creatine kinase, MB isoenzyme; DBP: diastolic blood pressure; hsCRP: high sensitivity C-reactive protein; LDL-C: low-density lipoprotein cholesterol; LVEF: left ventricular ejection fraction; MCH: mean corpuscular hemoglobin; MCHC: mean corpuscular hemoglobin concentration; MCV: mean corpuscular volume; NT-proBNP: N-terminal pro-B-type natriuretic peptide; SBP: systolic blood pressure.

## Data Availability

Data used to support the findings of this study are included within the article, and the original records of the enrolled patients are available from the corresponding authors upon reasonable request.

## Supplementary Materials

Supplementary Materials Supplementary Table 1: baseline characteristics according to the level of mean corpuscular volume (MCV) in the anemic and non-anemic group. Supplementary Table 2: baseline characteristics according to the level of mean corpuscular hemoglobin (MCH) in the anemic and nonanemic group. Supplementary Table 3: baseline characteristics according to the level of mean corpuscular hemoglobin concentration (MCHC) in the anemic and nonanemic group. Supplementary Figure 1: the prognostic values of MCV and MCH in UA/NSTEMI and STEMI patients. Supplementary Figure 2: the prognostic values of MCV and MCH in anemic and nonanemic patients with UA/NSTEMI.
