# Supplementary material for: Relationship between Red Blood Cell Indices (MCV, MCH, and MCHC) and Major Adverse Cardiovascular Events in Anemic and Nonanemic Patients with Acute Coronary Syndrome
Source: Dis Markers. 2022 Nov 3;2022:2193343. doi: 10.1155/2022/2193343 (PMC9649320; doi:10.1155/2022/2193343)
Supplement: Supplementary Materials — Supplementary Table 1: baseline characteristics according to the level of mean corpuscular volume (MCV) in the anemic and non-anemic group. Supplementary Table 2: baseline characteristics according to the level of mean corpuscular hemoglobin (MCH) in the anemic and nonanemic group. Supplementary Table 3: baseline characteristics according to the level of mean corpuscular hemoglobin concentration (MCHC) in the anemic and nonanemic group. Supplementary Figure 1: the prognostic values of MCV and MCH in UA/NSTEMI and STEMI patients. Supplementary Figure 2: the prognostic values of MCV and MCH in anemic and nonanemic patients with UA/NSTEMI. [file 2193343.f1.docx]

Supplementary Table 1. Baseline characteristics according to the level of mean corpuscular volume (MCV) in the anemic and nonanemic group

|  | **MCV (fl)** | | | | | | | |
| --- | --- | --- | --- | --- | --- | --- | --- | --- |
|  | **Anemic group (n=75)** | | |  | **Nonanemic group (n=318)** | | |  |
|  | Tertile 1  <91.3 (n=24) | Tertile 2  91.3-97.4 (n=27) | Tertile 3  >97.4 (n=24) |  | Tertile 1  <91.0 (n=104) | Tertile 2  91.0-95.0 (n=110) | Tertile 3  >95.0 (n=104) |  |
|  |  |  |  | p Value |  |  |  | p Value |
| **Demographics and risk factors** |  |  |  |  |  |  |  |  |
| Age, year | 64.3±11.6 | 66.4±9.6 | 68.3±9.9 | 0.420 | 58.0±9.6 | 59.0±9.7 | 60.0±9.8 | 0.339 |
| Male | 14 (58.3%) | 17 (63.0%) | 18 (75.0%) | 0.455 | 73 (70.2%) | 90 (81.8%) | 94 (90.4%) | 0.001 |
| Body mass index, kg/m² | 24.8±2.3 | 24.0±3.3 | 23.7±2.8 | 0.530 | 25.6±3.2 | 25.0±3.4 | 24.9±3.2 | 0.374 |
| SBP, mmHg | 120.0 (111.0-130.0) | 130.0 (120.0-140.0) | 122.5 (111.3-133.0) | 0.227 | 120.0 (110.5-137.5) | 120.0 (120.0-140.0) | 128.0 (120.0-138.0) | 0.979 |
| DBP, mmHg | 70.0 (61.0-80.0) | 78.0 (70.0-80.0) | 80.0 (67.0-80.0) | 0.451 | 80.0 (70.0-90.0) | 80.0 (70.0-80.0) | 80.0 (70.0-90.0) | 0.380 |
| Smoking | 7 (29.2%) | 11 (40.7%) | 15 (62.5%) | 0.061 | 49 (47.1%) | 71 (64.5%) | 71 (68.3%) | 0.004 |
| Diabetes | 13 (54.2%) | 6 (22.2%) | 3 (12.5%) | 0.004 | 27 (26.0%) | 21 (19.1%) | 13 (12.5%) | 0.048 |
| Family history | 9 (37.5%) | 8 (29.6%) | 8 (33.3%) | 0.838 | 45 (43.3%) | 41 (37.3%) | 52 (50.0%) | 0.172 |
| Past MI | 0 (0.0%) | 6 (22.2%) | 1 (4.2%) | 0.014 | 19 (18.3%) | 17 (15.5%) | 20 (19.2%) | 0.751 |
| Past PCI or CABG | 5 (20.8%) | 8 (29.6%) | 6 (25.0%) | 0.770 | 20 (19.2%) | 19 (17.3%) | 24 (23.1%) | 0.558 |
| Bleeding events | 4 (16.7%) | 1 (3.7%) | 0 (0.0%) | 0.051 | 3 (2.9%) | 2 (1.8%) | 4 (3.8%) | 0.670 |
| **Laboratory tests** |  |  |  |  |  |  |  |  |
| MCV, fl | 89.3 (87.8-90.5) | 93.6 (92.1-95.6) | 102.4 (98.4-104.5) | <0.001 | 88.6 (86.0-90.0) | 93.0 (91.7-93.9) | 97.7 (96.0-99.5) | <0.001 |
| MCH, pg | 29.2 (28.4-30.1) | 30.8 (30.1-31.6) | 32.4 (31.5-35.1) | <0.001 | 30.0 (29.3-30.6) | 31.3 (30.6-31.8) | 32.4 (31.6-33.6) | <0.001 |
| MCHC, g/L | 325.5 (318.5-335.0) | 325.0 (321.0-332.0) | 320.0 (315.0-336.5) | 0.741 | 340.0 (330.5-348.0) | 335.0 (329.3-343.8) | 330.0 (323.0-341.0) | 0.002 |
| LVEF, % | 72.0 (69.5-73.0) | 62.0 (49.0-66.0) | 54.5 (48.3-61.3) | 0.105 | 62.0 (52.0-67.5) | 62.5 (52.0-67.8) | 64.0 (56.0-68.5) | 0.714 |
| Aspartate aminotransferase, U/L | 27.7 (23.9-44.1) | 27.0 (15.8-82.5) | 33.2 (16.3-97.8) | 0.115 | 26.8 (17.8-50.6) | 28.6 (18.8-46.1) | 27.4 (19.1-45.0) | 0.955 |
| Alanine aminotransferase, U/L | 35.9 (16.0-49.0) | 21.3 (14.5-78.3) | 19.0 (14.3-33.0) | 0.919 | 25.1 (19.5-51.9) | 34.3 (18.6-62.3) | 23.3 (16.0-38.1) | 0.346 |
| Creatinine, μmol/L | 68.7 (57.3-75.7) | 59.3 (53.2-69.1) | 63.9 (56.6-79.9) | 0.572 | 68.1 (57.4-77.1) | 64.4 (57.2-69.8) | 68.4 (62.3-77.0) | 0.145 |
| LDL-C, mmol/L | 2.1 (1.9-3.3) | 1.5 (1.3-2.5) | 1.9 (1.4-2.3) | 0.397 | 2.3 (1.7-2.8) | 2.1 (1.7-2.6) | 2.5 (1.8-3.2) | 0.948 |
| CKMB, U/L | 12.7 (11.8-28.5) | 13.6 (9.1-20.2) | 20.6 (9.2-85.4) | 0.447 | 13.9 (11.1-18.4) | 17.0 (12.4-32.9) | 14.9 (12.6-22.1) | 0.418 |
| Homocysteine, μmol/L | 20.2 (11.4-28.6) | 13.5 (10.9-17.8) | 14.4 (11.2-57.1) | 0.884 | 14.0 (11.6-18.3) | 14.5 (12.3-20.8) | 21.0 (12.7-39.6) | <0.001 |
| RBC,10^12/L | 3.9 (2.7-5.5) | 4.0 (3.2-14.3) | 6.2 (3.1-7.3) | 0.974 | 4.6 (3.9-6.3) | 5.0 (4.0-7.1) | 4.3 (3.6-5.4) | 0.307 |
| Hemoglobin, g/L | 112.9±11.4 | 115.0±14.4 | 114.0±14.0 | 0.844 | 142.7±11.9 | 145.0±12.4 | 146.5±12.2 | 0.083 |
| HCT, % | 34.8±2.9 | 35.1±4.2 | 35.0±4.4 | 0.947 | 42.2±3.1 | 43.1±3.3 | 44.0±3.5 | 0.001 |
| RDW-CV, % | 14.1 (13.0-15.0) | 13.1 (12.3-13.5) | 14.1 (12.9-15.0) | 0.042 | 13.1 (12.6-13.5) | 13.0 (12.6-13.5) | 13.1 (12.7-13.9) | 0.055 |
| hsCRP, mg/dL | 1.3 (0.7-2.4) | 1.2 (0.4-3.0) | 3.0 (1.3-6.1) | 0.057 | 1.6 (0.8-4.1) | 1.7 (0.8-3.9) | 1.1 (0.7-2.9) | 0.456 |
| NT-proBNP, pg/mL | 139.6 (63.8-467.3) | 467.0 (211.9-916.0) | 204.2 (167.6-1304.8) | 0.705 | 237.3 (116.2-629.3) | 304.3 (80.2-1002.9) | 206.7 (86.0-941.4) | 0.228 |
| **Primary diagnosis at admission** |  |  |  |  |  |  |  |  |
| UA | 19 (79.2%) | 21 (77.8%) | 19 (79.2%) |  | 77 (74.0%) | 80 (72.7%) | 77 (74.0%) |  |
| NSTEMI | 1 (4.2%) | 2 (7.4%) | 1 (4.2%) |  | 13 (12.5%) | 10 (9.1%) | 9 (8.7%) |  |
| STEMI | 4 (16.7%) | 4 (14.8%) | 4 (16.7%) |  | 14 (13.5%) | 20 (18.2%) | 18 (17.3%) |  |
| **In-hospital treatment** |  |  |  |  |  |  |  |  |
| Coronary revascularization | 24 (100.0%) | 27 (100.0%) | 23 (95.8%) | 0.341 | 102 (98.1%) | 108 (98.2%) | 103 (99.0%) | 0.828 |
| Number of lesion vessels | 3.0 (2.0-3.0) | 3.0 (3.0-4.0) | 3.0 (2.0-3.0) | 0.996 | 3.0 (2.0-3.0) | 2.0 (2.0-3.0) | 3.0 (2.0-3.0) | 0.007 |
| Number of stents | 2.0 (1.0-3.0) | 2.0 (1.0-3.0) | 2.0 (1.0-3.0) | 0.854 | 2.0 (1.0-3.0) | 2.0 (1.0-3.0) | 2.0 (1.0-3.0) | 0.051 |
| **Discharge medication** |  |  |  |  |  |  |  |  |
| Aspirin | 24 (100.0%) | 27 (100.0%) | 22 (91.7%) | 0.113 | 104 (100.0%) | 110 (100.0%) | 102 (98.1%) | 0.126 |
| Clopidogrel | 22 (91.7%) | 26 (96.3%) | 24 (100.0%) | 0.336 | 102 (98.1%) | 108 (98.2%) | 103 (99.0%) | 0.828 |
| Ticagrelor | 4 (16.7%) | 1 (3.7%) | 0 (0.0%) | 0.051 | 5 (4.8%) | 5 (4.5%) | 4 (3.8%) | 0.941 |
| Beta-blocker | 20 (83.3%) | 22 (81.5%) | 22 (91.7%) | 0.558 | 92 (88.5%) | 98 (89.1%) | 92 (88.5) | 0.986 |
| ACEI/ARB | 21 (87.5%) | 24 (88.9%) | 19 (79.2%) | 0.579 | 97 (93.3%) | 102 (92.7%) | 96 (92.3%) | 0.965 |
| Statin | 24 (100.0%) | 27 (100.0%) | 24 (100.0%) |  | 103 (99.0%) | 106 (96.4%) | 102 (98.1%) | 0.400 |
| Calcium channel blocker | 5 (20.8%) | 8 (29.6%) | 6 (25.0%) | 0.770 | 30 (28.8%) | 20 (18.2%) | 20 (19.2%) | 0.120 |
| **MACEs** | 7 (29.2%) | 5 (18.5%) | 8 (33.3%) | 0.463 | 10 (9.6%) | 12 (10.9%) | 24 (23.1%) | 0.009 |
| All-cause death | 4 (16.7%) | 2 (7.4%) | 3 (12.5%) | 0.595 | 3 (2.9%) | 2 (1.8%) | 3 (2.9%) | 0.846 |
| Cardiac death | 3 (12.5%) | 2 (7.4%) | 2 (8.3%) | 0.806 | 2 (1.9%) | 1 (0.9%) | 1 (1.0%) | 0.759 |
| Acute myocardial infarction | 1 (4.2%) | 1 (3.7%) | 1 (4.2%) | 0.995 | 1 (1.0%) | 1 (0.9%) | 1 (1.0%) | 0.999 |
| Urgent revascularization | 0 (0.0%) | 1 (3.7%) | 2 (8.3%) | 0.336 | 5 (4.8%) | 3 (2.7%) | 7 (6.7%) | 0.385 |
| Re-hospitalization | 4 (16.7%) | 2 (7.4%) | 4 (16.7%) | 0.527 | 2 (1.9%) | 7 (6.4%) | 10 (9.6%) | 0.063 |
| Cerebrovascular diseases | 1 (4.2%) | 0 (0.0%) | 1 (4.2%) | 0.561 | 1 (1.0%) | 1 (0.9%) | 5 (4.8%) | 0.087 |

Data are shown as mean ± SD, median (interquartile range) or number (%). ACEI: angiotensin converting enzyme inhibitor; ARB: angiotensin receptor blocker; CABG: coronary artery bypass grafting; CKMB: creatine kinase, MB isoenzyme; DBP, diastolic blood pressure; HCT: hematocrit value; hsCRP: high sensitivity C-reactive protein; LDL-C: low density lipoprotein cholesterol; LVEF: left ventricular ejection fraction; MACEs: major adverse cardiovascular events; MCH: mean corpuscular hemoglobin; MCHC: mean corpuscular hemoglobin concentration; MCV: mean corpuscular volume; MI: myocardial infarction; NSTEMI: non-ST elevated myocardial infarction; NT-proBNP: N-terminal pro-B-type natriuretic peptide; PCI: percutaneous coronary intervention; RBC: red blood cell; RDW-CV: red cell volume distribution width-coefficient of variation; SBP, systolic blood pressure; STEMI: ST elevated myocardial infarction; UA: unstable angina.

Supplementary Table 2. Baseline characteristics according to the level of mean corpuscular hemoglobin (MCH) in the anemic and nonanemic group

|  | **MCH (pg)** | | | | | | | |
| --- | --- | --- | --- | --- | --- | --- | --- | --- |
|  | **Anemic group (n=75)** | | |  | **Nonanemic group (n=318)** | | |  |
|  | Tertile 1  <29.7 (n=24) | Tertile 2  29.7-31.7 (n=26) | Tertile 3  >31.7 (n=25) |  | Tertile 1  <30.6 (n=101) | Tertile 2  30.6-31.9 (n=117) | Tertile 3  >31.9 (n=100) |  |
|  |  |  |  | p Value |  |  |  | p Value |
| **Demographics and risk factors** |  |  |  |  |  |  |  |  |
| Age, year | 66.0±11.4 | 64.3±9.8 | 68.8±9.6 | 0.300 | 59.9±9.2 | 59.1±10.1 | 58.0±9.8 | 0.386 |
| Male | 13 (54.2%) | 16 (61.5%) | 20 (80.0%) | 0.145 | 63 (62.4%) | 98 (83.8%) | 96 (96.0%) | <0.001 |
| Body mass index, kg/m² | 24.0±2.5 | 24.4±3.3 | 24.0±2.6 | 0.864 | 25.6±3.5 | 24.9±3.1 | 25.1±3.2 | 0.366 |
| SBP, mmHg | 120.0 (100.0-130.0) | 124.0 (120.0-138.0) | 130.0 (119.0-138.0) | 0.296 | 120.0 (110.0-132.5) | 120.0 (120.0-138.5) | 130.0 (120.0-141.0) | 0.725 |
| DBP, mmHg | 70.0 (60.0-80.0) | 78.0 (70.0-80.0) | 80.0 (68.0-81.5) | 0.264 | 80.0 (70.0-86.0) | 80.0 (70.0-84.5) | 80.0 (79.0-90.0) | 0.105 |
| Smoking | 8 (33.3%) | 11 (42.3%) | 14 (56.0%) | 0.273 | 40 (39.6%) | 74 (63.2%) | 77 (77.0%) | <0.001 |
| Diabetes | 10 (41.7%) | 10 (38.5%) | 2 (8.0%) | 0.016 | 29 (28.7%) | 18 (15.4%) | 14 (14.0%) | 0.013 |
| Family history | 8 (33.3%) | 10 (38.5%) | 7 (28.0%) | 0.731 | 43 (42.6%) | 50 (42.7%) | 45 (45.0%) | 0.926 |
| Past MI | 1 (4.2%) | 3 (11.5%) | 3 (12.0%) | 0.572 | 21 (20.8%) | 16 (13.7%) | 19 (19.0%) | 0.352 |
| Past PCI or CABG | 6 (25.0%) | 7 (26.9%) | 6 (24.0%) | 0.971 | 19 (18.8%) | 25 (21.4%) | 19 (19.0%) | 0.868 |
| Bleeding events | 4 (16.7%) | 1 (3.8%) | 0 (0.0%) | 0.050 | 2 (2.0%) | 2 (1.7%) | 5 (5.0%) | 0.285 |
| **Laboratory tests** |  |  |  |  |  |  |  |  |
| MCV, fl | 89.3 (88.0-90.6) | 93.6 (90.8-97.3) | 102.5 (97.1-104.5) | <0.001 | 88.9 (86.0-90.5) | 93.8 (91.0-95.8) | 98.4 (95.5-100.6) | <0.001 |
| MCH, pg | 28.9 (28.2-29.2) | 30.7 (30.1-31.2) | 32.6 (31.8-35.8) | <0.001 | 29.8 (29.1-30.2) | 31.3 (30.8-31.7) | 33.1 (32.6-33.9) | <0.001 |
| MCHC, g/L | 322.0 (317.0-324.0) | 325.0 (320.0-332.0) | 331.0 (317.0-349.0) | 0.001 | 333.0 (327.5-340.0) | 333.5 (326.3-343.8) | 342.0 (331.5-346.5) | <0.001 |
| LVEF, % | 73.0 (63.0-73.0) | 66.0 (58.0-70.0) | 53.0 (45.5-61.0) | 0.149 | 62.0 (50.0-67.5) | 63.0 (55.0-68.0) | 65.0 (56.0-68.0) | 0.611 |
| Aspartate aminotransferase, U/L | 27.8 (17.6-43.1) | 27.0 (15.8-84.1) | 36.5 (18.1-87.6) | 0.127 | 25.6 (17.9-47.1) | 28.1 (18.9-50.0) | 27.4 (19.6-40.5) | 0.442 |
| Alanine aminotransferase, U/L | 17.8 (14.8-33.8) | 23.2 (14.5-58.7) | 30.8 (14.5-52.4) | 0.210 | 21.5 (18.0-37.0) | 32.9 (21.1-65.4) | 26.4 (16.0-41.2) | 0.014 |
| Creatinine, μmol/L | 68.9 (56.7-78.0) | 62.7 (53.2-69.1) | 65.1 (56.1-75.4) | 0.875 | 66.7 (56.5-73.4) | 67.0 (57.8-76.8) | 66.4 (62.7-75.6) | 0.109 |
| LDL-C, mmol/L | 2.5 (1.6-3.9) | 2.1 (1.5-2.5) | 1.7 (1.1-2.0) | 0.019 | 2.3 (1.7-2.9) | 2.3 (1.7-2.8) | 2.1 (1.8-3.0) | 0.776 |
| CKMB, U/L | 12.6 (11.3-34.5) | 12.6 (9.4-26.5) | 17.2 (11.6-59.2) | 0.230 | 14.8 (11.9-34.4) | 14.3 (11.1-19.4) | 15.0 (12.6-25.6) | 0.449 |
| Homocysteine, μmol/L | 23.3 (11.8-28.0) | 14.9 (10.3-24.5) | 13.4 (10.8-35.2) | 0.266 | 13.4 (11.6-19.3) | 14.5 (12.0-21.1) | 21.1 (15.1-48.3) | <0.001 |
| RBC,10^12/L | 5.2 (4.0-6.6) | 3.4 (2.9-7.3) | 5.9 (3.8-7.5) | 0.303 | 4.7 (4.0-6.0) | 4.6 (3.7-6.6) | 4.3 (3.5-5.6) | 0.761 |
| Hemoglobin, g/L | 111.1±11.8 | 115.5±13.7 | 115.1±14.0 | 0.441 | 140.5±12.4 | 144.9±10.2 | 148.9±13.0 | <0.001 |
| HCT, % | 34.8±3.4 | 35.3±4.2 | 34.7±4.1 | 0.834 | 42.5±3.6 | 43.1±3.0 | 43.7±3.5 | 0.052 |
| RDW-CV, % | 14.6 (13.5-15.2) | 13.4 (12.4-14.1) | 12.9 (12.7-14.6) | 0.054 | 13.2 (12.9-13.6) | 13.0 (12.6-13.7) | 13.1 (12.6-13.8) | 0.015 |
| hsCRP, mg/dL | 1.5 (1.0-4.7) | 1.2 (0.5-2.8) | 2.6 (1.2-5.6) | 0.444 | 1.9 (0.8-4.2) | 1.2 (0.7-3.6) | 1.2 (0.9-2.7) | 0.388 |
| NT-proBNP, pg/mL | 193.8 (70.0-616.5) | 211.9 (132.9-679.1) | 783.9 (197.3-1332.5) | 0.120 | 237.3 (119.7-671.8) | 200.0 (72.4-848.3) | 379.6 (117.9-956.3) | 0.662 |
| **Primary diagnosis at admission** |  |  |  |  |  |  |  |  |
| UA | 18 (75.0%) | 22 (84.6%) | 19 (76.0%) |  | 72 (71.3%) | 92 (78.6%) | 70 (70.0%) |  |
| NSTEMI | 1 (4.2%) | 2 (7.7%) | 1 (4.0%) |  | 14 (13.9%) | 7 (6.0%) | 11 (11.0%) |  |
| STEMI | 5 (20.8%) | 2 (7.7%) | 5 (20.0%) |  | 15 (14.9%) | 18 (15.4%) | 19 (19.0%) |  |
| **In-hospital treatment** |  |  |  |  |  |  |  |  |
| Coronary revascularization | 24 (100.0%) | 26 (100.0%) | 24 (96.0%) | 0.363 | 99 (98.0%) | 114 (97.4%) | 100 (100.0%) | 0.294 |
| Number of lesion vessels | 3.0 (2.0-3.0) | 3.0 (2.0-3.0) | 3.0 (2.0-3.0) | 0.722 | 3.0 (2.0-3.0) | 2.0 (2.0-3.0) | 3.0 (1.3-3.0) | 0.037 |
| Number of stents | 2.0 (1.0-3.0) | 2.0 (1.0-3.3) | 2.0 (1.0-3.0) | 0.794 | 3.0 (1.0-3.0) | 2.0 (1.0-3.0) | 2.0 (1.0-3.0) | 0.097 |
| **Discharge medication** |  |  |  |  |  |  |  |  |
| Aspirin | 24 (100.0%) | 25 (96.2%) | 24 (96.0%) | 0.616 | 101 (100.0%) | 117 (100.0%) | 98 (98.0%) | 0.111 |
| Clopidogrel | 22 (91.7%) | 25 (96.2%) | 25 (100.0%) | 0.330 | 99 (98.0%) | 116 (99.1%) | 98 (98.0%) | 0.735 |
| Ticagrelor | 4 (16.7%) | 1 (3.8%) | 0 (0.0%) | 0.050 | 6 (5.9%) | 4 (3.4%) | 4 (4.0%) | 0.646 |
| Beta-blocker | 18 (75.0%) | 22 (84.6%) | 24 (96.0%) | 0.115 | 89 (88.1%) | 100 (85.5%) | 93 (93.0%) | 0.213 |
| ACEI/ARB | 20 (83.3%) | 21 (80.8%) | 23 (92.0%) | 0.497 | 95 (94.1%) | 107 (91.5%) | 93 (93.0%) | 0.756 |
| Statin | 24 (100.0%) | 26 (100.0%) | 25 (100.0%) |  | 100 (99.0%) | 114 (97.4%) | 97 (97.0%) | 0.590 |
| Calcium channel blocker | 6 (25.0%) | 6 (23.1%) | 7 (28.0%) | 0.921 | 30 (29.7%) | 22 (18.8%) | 18 (18.0%) | 0.077 |
| **MACEs** | 6 (25.0%) | 6 (23.1%) | 8 (32.0%) | 0.752 | 10 (9.9%) | 13 (11.1%) | 23 (23.0%) | 0.013 |
| All-cause death | 4 (16.7%) | 1 (3.8%) | 4 (16.0%) | 0.285 | 2 (2.0%) | 2 (1.7%) | 4 (4.0%) | 0.515 |
| Cardiac death | 3 (12.5%) | 1 (3.8%) | 3 (12.0%) | 0.492 | 2 (2.0%) | 1 (0.9%) | 1 (1.0%) | 0.729 |
| Acute myocardial infarction | 1 (4.2%) | 1 (3.8%) | 1 (4.0%) | 0.998 | 1 (1.0%) | 2 (1.7%) | 0 (0.0%) | 0.430 |
| Urgent revascularization | 0 (0.0%) | 1 (3.8%) | 2 (8.0%) | 0.360 | 3 (3.0%) | 6 (5.1%) | 6 (6.0%) | 0.578 |
| Re-hospitalization | 3 (12.5%) | 4 (15.4%) | 3 (12.0%) | 0.929 | 4 (4.0%) | 5 (4.3%) | 10 (10.0%) | 0.121 |
| Cerebrovascular diseases | 1 (4.2%) | 0 (0.0%) | 1 (4.0%) | 0.579 | 1 (1.0%) | 2 (1.7%) | 4 (4.0%) | 0.313 |

Data are shown as mean ± SD, median (interquartile range) or number (%). ACEI: angiotensin converting enzyme inhibitor; ARB: angiotensin receptor blocker; CABG: coronary artery bypass grafting; CKMB: creatine kinase, MB isoenzyme; DBP, diastolic blood pressure; HCT: hematocrit value; hsCRP: high sensitivity C-reactive protein; LDL-C: low density lipoprotein cholesterol; LVEF: left ventricular ejection fraction; MACEs: major adverse cardiovascular events; MCH: mean corpuscular hemoglobin; MCHC: mean corpuscular hemoglobin concentration; MCV: mean corpuscular volume; MI: myocardial infarction; NSTEMI: non-ST elevated myocardial infarction; NT-proBNP: N-terminal pro-B-type natriuretic peptide; PCI: percutaneous coronary intervention; RBC: red blood cell; RDW-CV: red cell volume distribution width-coefficient of variation; SBP, systolic blood pressure; STEMI: ST elevated myocardial infarction; UA: unstable angina.

Supplementary Table 3. Baseline characteristics according to the level of mean corpuscular hemoglobin concentration (MCHC) in the anemic and nonanemic group

|  | **MCHC (g/L)** | | | | | | | |
| --- | --- | --- | --- | --- | --- | --- | --- | --- |
|  | **Anemic group (n=75)** | | |  | **Nonanemic group (n=318)** | | |  |
|  | Tertile 1  <320.3 (n=25) | Tertile 2  320.3-328.0 (n=26) | Tertile 3  >328.0 (n=24) |  | Tertile 1  <330.0 (n=94) | Tertile 2  330.0-341.0 (n=122) | Tertile 3  >341.0 (n=102) |  |
|  |  |  |  | p Value |  |  |  | p Value |
| **Demographics and risk factors** |  |  |  |  |  |  |  |  |
| Age, year | 68.3±11.0 | 63.4±10.4 | 67.5±9.2 | 0.200 | 61.0±10.1 | 59.1±9.1 | 57.1±9.9 | 0.017 |
| Male | 15 (60.0%) | 18 (69.2%) | 16 (66.7%) | 0.776 | 65 (69.1%) | 96 (78.7%) | 96 (94.1%) | <0.001 |
| Body mass index, kg/m² | 23.5±3.1 | 24.7±2.3 | 24.3±3.0 | 0.430 | 25.3±3.4 | 25.1±3.1 | 25.2±3.4 | 0.917 |
| SBP, mmHg | 120.0 (120.0-136.0) | 122.0 (110.5-132.0) | 130.0 (117.8-140.0) | 0.220 | 125.0 (120.0-135.0) | 126.5 (120.0-140.0) | 120.0 (116.0-140.0) | 0.829 |
| DBP, mmHg | 80.0 (70.0-80.0) | 72.0 (69.5-80.0) | 70.0 (61.5-80.0) | 0.555 | 80.0 (70.0-90.0) | 80.0 (71.3-83.8) | 80.0 (70.0-88.0) | 0.263 |
| Smoking | 9 (36.0%) | 13 (50.0%) | 11 (45.8%) | 0.588 | 48 (51.1%) | 73 (59.8%) | 70 (68.6%) | 0.043 |
| Diabetes | 7 (28.0%) | 9 (34.6%) | 6 (25.0%) | 0.745 | 17 (18.1%) | 24 (19.7%) | 20 (19.6%) | 0.949 |
| Family history | 9 (36.0%) | 7 (26.9%) | 9 (37.5%) | 0.688 | 42 (44.7%) | 53 (43.4%) | 43 (42.2%) | 0.938 |
| Past MI | 0 (0.0%) | 4 (15.4%) | 3 (12.5%) | 0.136 | 18 (19.1%) | 23 (18.9%) | 15 (14.7%) | 0.645 |
| Past PCI or CABG | 5 (20.0%) | 8 (30.8%) | 6 (25.0%) | 0.676 | 22 (23.4%) | 24 (19.7%) | 17 (16.7%) | 0.497 |
| Bleeding events | 3 (12.0%) | 2 (7.7%) | 0 (0.0%) | 0.234 | 2 (2.1%) | 2 (1.6%) | 5 (4.9%) | 0.303 |
| **Laboratory tests** |  |  |  |  |  |  |  |  |
| MCV, fl | 97.5 (90.6-102.2) | 91.8 (90.1-94.6) | 93.2 (90.2-103.8) | 0.696 | 95.4 (90.7-97.8) | 92.9 (89.4-95.6) | 91.4 (87.8-95.5) | 0.004 |
| MCH, pg | 31.2 (28.9-31.8) | 29.9 (29.2-30.5) | 31.7 (30.6-35.1) | 0.001 | 30.7 (29.7-31.6) | 31.1 (30.2-31.9) | 31.8 (30.7-33.5) | <0.001 |
| MCHC, g/L | 317.0 (315.0-319.0) | 324.5 (321.8-327.0) | 342.0 (332.0-348.5) | <0.001 | 324.0 (321.0-328.0) | 335.0 (333.0-338.0) | 347.0 (344.0-351.0) | <0.001 |
| LVEF, % | 66.0 (56.0-72.0) | 63.0 (46.8-73.0) | 61.5 (51.0-70.3) | 0.297 | 64.0 (60.5-69.0) | 60.0 (48.0-67.8) | 63.0 (55.0-67.0) | 0.195 |
| Aspartate aminotransferase, U/L | 29.8 (24.1-47.3) | 27.0 (15.3-81.8) | 27.0 (18.5-82.9) | 0.786 | 27.8 (18.6-53.0) | 25.9 (18.2-39.3) | 27.4 (17.4-40.5) | 0.397 |
| Alanine aminotransferase, U/L | 16.9 (14.3-30.8) | 30.5 (16.0-53.9) | 35.1 (14.2-86.8) | 0.162 | 26.4 (18.2-42.0) | 24.2 (17.7-46.6) | 31.9 (18.8-55.0) | 0.021 |
| Creatinine, μmol/L | 59.9 (57.2-78.8) | 59.3 (54.3-73.4) | 68.8 (61.5-72.7) | 0.434 | 66.7 (55.7-73.1) | 70.2 (62.4-78.4) | 64.1 (59.4-70.3) | 0.693 |
| LDL-C, mmol/L | 2.0 (1.5-2.5) | 2.2 (1.6-3.2) | 1.8 (1.3-2.1) | 0.074 | 2.4 (1.8-3.0) | 2.3 (1.7-2.8) | 2.2 (1.8-2.8) | 0.402 |
| CKMB, U/L | 15.2 (10.2-54.2) | 12.2 (9.4-33.3) | 14.4 (11.8-25.6) | 0.083 | 15.8 (12.6-44.2) | 15.0 (11.1-22.5) | 14.7 (11.3-17.6) | 0.924 |
| Homocysteine, μmol/L | 16.2 (11.6-23.3) | 13.0 (11.4-25.4) | 14.8 (10.8-39.9) | 0.480 | 14.5 (11.7-27.9) | 16.0 (13.0-21.4) | 15.0 (11.9-22.5) | 0.483 |
| RBC,10^12/L | 5.2 (2.9-6.6) | 5.3 (3.1-14.5) | 4.0 (3.5-7.4) | 0.963 | 4.9 (3.9-6.8) | 4.3 (3.5-6.5) | 4.6 (3.8-5.8) | 0.063 |
| Hemoglobin, g/L | 109.7±13.5 | 113.7±14.1 | 118.7±10.6 | 0.056 | 140.5±12.1 | 143.2±10.5 | 150.5±12.3 | <0.001 |
| HCT, % | 34.9±4.2 | 35.0±4.4 | 34.9±3.0 | 0.988 | 43.5±3.7 | 42.7±3.0 | 43.2±3.4 | 0.179 |
| RDW-CV, % | 14.2 (13.4-15.1) | 13.4 (12.8-14.2) | 12.9 (12.3-13.8) | 0.001 | 13.5 (13.0-13.9) | 13.0 (12.6-13.4) | 12.9 (12.4-13.5) | <0.001 |
| hsCRP, mg/dL | 1.6 (1.0-3.9) | 1.0 (0.3-3.4) | 2.4 (1.2-3.8) | 0.417 | 1.0 (0.5-3.6) | 1.6 (0.9-3.2) | 1.5 (0.9-4.0) | 0.486 |
| NT-proBNP, pg/mL | 334.7 (179.1-906.3) | 163.4 (45.2-985.3) | 341.8 (184.9-816.9) | 0.967 | 192.1 (66.4-816.4) | 432.6 (155.3-1030.5) | 228.2 (108.5-454.1) | 0.010 |
| **Primary diagnosis at admission** |  |  |  |  |  |  |  |  |
| UA | 19 (76.0%) | 22 (84.6%) | 18 (75.0%) |  | 70 (74.5%) | 89 (73.0%) | 75 (73.5%) |  |
| NSTEMI | 0 (0.0%) | 1 (3.8%) | 3 (12.5%) |  | 12 (12.8%) | 11 (9.0%) | 9 (8.8%) |  |
| STEMI | 6 (24.0%) | 3 (11.5%) | 3 (12.5%) |  | 12 (12.8%) | 22 (18.0%) | 18 (17.6%) |  |
| **In-hospital treatment** |  |  |  |  |  |  |  |  |
| Coronary revascularization | 25 (100.0%) | 26 (100.0%) | 23 (95.8%) | 0.341 | 92 (97.9%) | 120 (98.4%) | 101 (99.0%) | 0.810 |
| Number of lesion vessels | 3.0 (2.0-3.0) | 2.5 (2.0-3.0) | 3.0 (2.0-3.0) | 0.881 | 3.0 (2.0-3.0) | 3.0 (2.0-3.0) | 3.0 (2.0-3.0) | 0.315 |
| Number of stents | 2.0 (1.0-3.0) | 2.0 (1.0-3.0) | 2.0 (1.0-3.8) | 0.716 | 2.0 (1.0-3.0) | 2.0 (1.0-3.0) | 2.0 (1.8-3.0) | 0.046 |
| **Discharge medication** |  |  |  |  |  |  |  |  |
| Aspirin | 24 (96.0%) | 26 (100.0%) | 23 (95.8%) | 0.579 | 92 (97.9%) | 122 (100.0%) | 102 (100.0%) | 0.091 |
| Clopidogrel | 25 (100.0%) | 25 (96.2%) | 22 (91.7%) | 0.330 | 94 (100.0%) | 118 (96.7%) | 101 (99.0%) | 0.133 |
| Ticagrelor | 1 (4.0%) | 2 (7.7%) | 2 (8.3%) | 0.804 | 4 (4.3%) | 8 (6.6%) | 2 (2.0%) | 0.247 |
| Beta-blocker | 22 (88.0%) | 20 (76.9%) | 22 (91.7%) | 0.304 | 83 (88.3%) | 102 (83.6%) | 97 (95.1%) | 0.026 |
| ACEI/ARB | 17 (68.0%) | 23 (88.5%) | 24 (100.0%) | 0.006 | 87 (92.6%) | 113 (92.6%) | 95 (93.1%) | 0.985 |
| Statin | 25 (100.0%) | 26 (100.0%) | 24 (100.0%) |  | 93 (98.9%) | 118 (96.7%) | 100 (98.0%) | 0.535 |
| Calcium channel blocker | 6 (24.0%) | 4 (15.4%) | 9 (37.5%) | 0.196 | 22 (23.4%) | 24 (19.7%) | 24 (23.5%) | 0.729 |
| **MACEs** | 6 (24.0%) | 8 (30.8%) | 6 (25.0%) | 0.840 | 16 (17.0%) | 18 (14.8%) | 12 (11.8%) | 0.575 |
| All-cause death | 2 (8.0%) | 4 (15.4%) | 3 (12.5%) | 0.717 | 4 (4.3%) | 1 (0.8%) | 3 (2.9%) | 0.264 |
| Cardiac death | 0 (0.0%) | 4 (15.4%) | 3 (12.5%) | 0.136 | 3 (3.2%) | 1 (0.8%) | 0 (0.0%) | 0.115 |
| Acute myocardial infarction | 1 (4.0%) | 2 (7.7%) | 0 (0.0%) | 0.382 | 1 (1.1%) | 1 (0.8%) | 1 (1.0%) | 0.982 |
| Urgent revascularization | 0 (0.0%) | 2 (7.7%) | 1 (4.2%) | 0.374 | 4 (4.3%) | 7 (5.7%) | 4 (3.9%) | 0.790 |
| Re-hospitalization | 6 (24.0%) | 2 (7.7%) | 2 (8.3%) | 0.157 | 5 (5.3%) | 9 (7.4%) | 5 (4.9%) | 0.702 |
| Cerebrovascular diseases | 1 (4.0%) | 1 (3.8%) | 0 (0.0%) | 0.616 | 3 (3.2%) | 3 (2.5%) | 1 (1.0%) | 0.557 |

Data are shown as mean ± SD, median (interquartile range) or number (%). ACEI: angiotensin converting enzyme inhibitor; ARB: angiotensin receptor blocker; CABG: coronary artery bypass grafting; CKMB: creatine kinase, MB isoenzyme; DBP, diastolic blood pressure; HCT: hematocrit value; hsCRP: high sensitivity C-reactive protein; LDL-C: low density lipoprotein cholesterol; LVEF: left ventricular ejection fraction; MACEs: major adverse cardiovascular events; MCH: mean corpuscular hemoglobin; MCHC: mean corpuscular hemoglobin concentration; MCV: mean corpuscular volume; MI: myocardial infarction; NSTEMI: non-ST elevated myocardial infarction; NT-proBNP: N-terminal pro-B-type natriuretic peptide; PCI: percutaneous coronary intervention; RBC: red blood cell; RDW-CV: red cell volume distribution width-coefficient of variation; SBP, systolic blood pressure; STEMI: ST elevated myocardial infarction; UA: unstable angina.


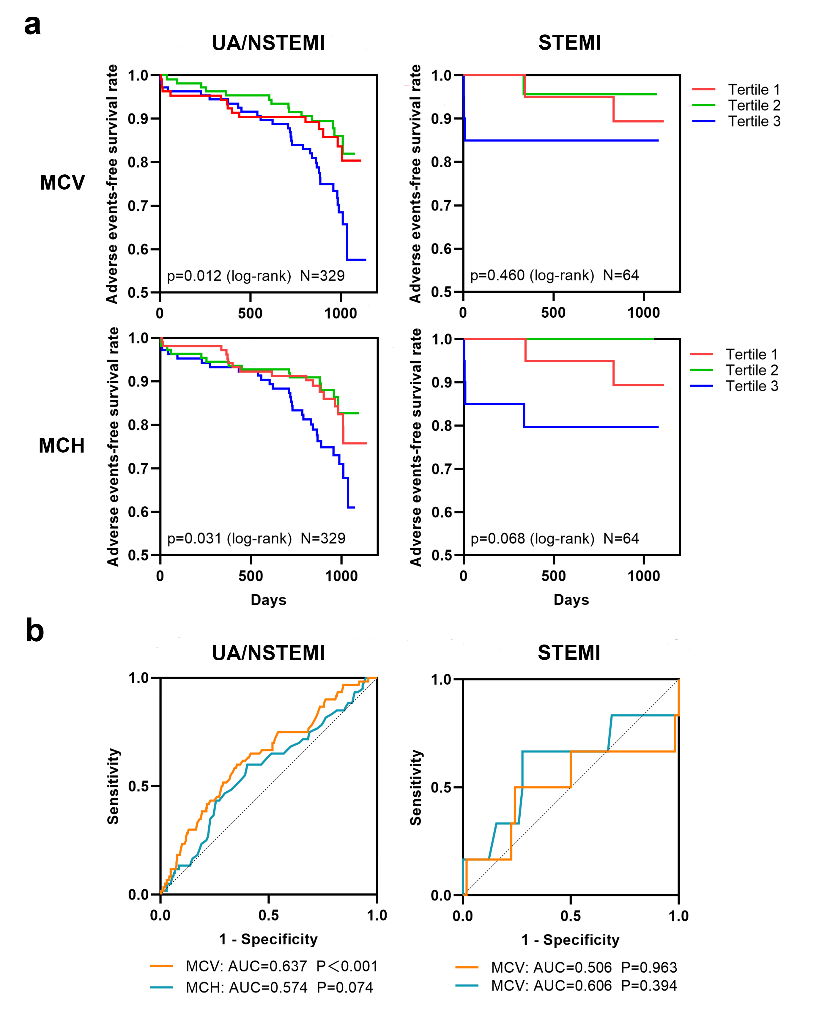


Supplementary Figure 1: The prognostic values of MCV and MCH in UA/NSTEMI and STEMI patients. (a) Adverse events free survival rate for MACEs in UA/NSTEMI and STEMI patients divided by tertiles of MCV and MCH. (b) ROC curve analysis of MCV and MCH in UA/NSTEMI and STEMI patients. AUC: area under the curve; MACEs: major adverse cardiovascular events; MCH: mean corpuscular hemoglobin; MCV: mean corpuscular volume; NSTEMI: non-ST elevated myocardial infarction; STEMI: ST elevated myocardial infarction; UA: unstable angina.


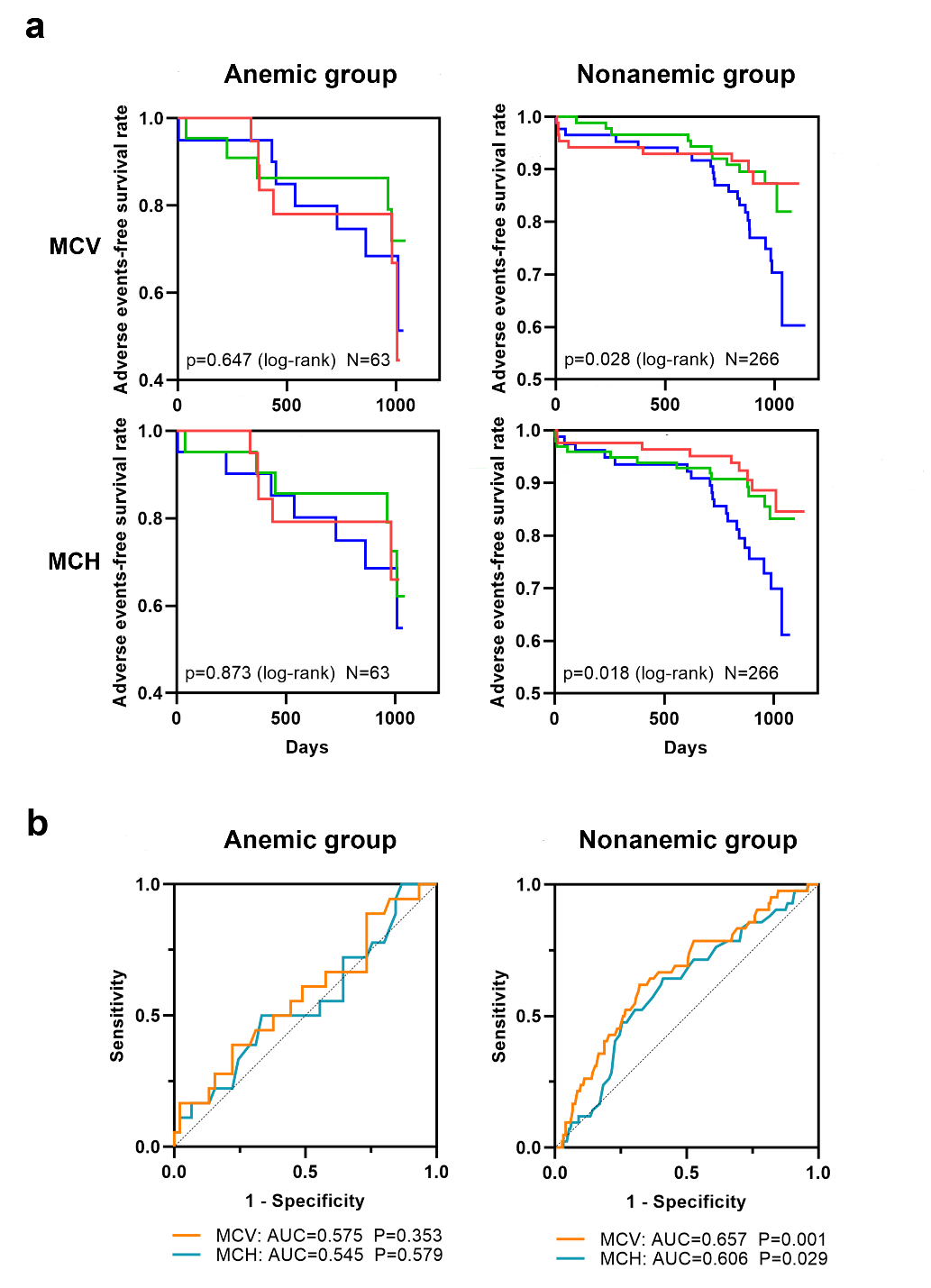


Supplementary Figure 2: The prognostic values of MCV and MCH in anemic and nonanemic patients with UA/NSTEMI. (a) Adverse events free survival rate for MACEs in UA/NSTEMI patients with or without anemia. (b) ROC curve analysis of MCV and MCH in the anemic and non-anemic group of UA/NSTEMI patients. AUC: area under the curve; MACEs: major adverse cardiovascular events; MCH: mean corpuscular hemoglobin; MCV: mean corpuscular volume; NSTEMI: non-ST elevated myocardial infarction; STEMI: ST elevated myocardial infarction; UA: unstable angina.
